# Lake and Sea Populations of *Mysis relicta* (Crustacea, Mysida) with Different Visual-Pigment Absorbance Spectra Use the Same A1 Chromophore

**DOI:** 10.1371/journal.pone.0088107

**Published:** 2014-02-07

**Authors:** Nikolai Belikov, Marina Yakovleva, Tatiana Feldman, Olga Demina, Andrei Khodonov, Magnus Lindström, Kristian Donner, Mikhail Ostrovsky

**Affiliations:** 1 Institute of Biochemical Physics, Russian Academy of Sciences, Moscow, Russia; 2 Biological Faculty, Moscow State University, Moscow, Russia; 3 Tvärminne Zoological Station, University of Helsinki, Hanko, Finland; 4 Department of Biosciences, University of Helsinki, Helsinki, Finland; The Australian National University, Australia

## Abstract

Glacial-relict species of the genus *Mysis* (opossum shrimps) inhabiting both fresh-water lakes and brackish sea waters in northern Europe show a consistent lake/sea dichotomy in eye spectral sensitivity. The absorbance peak (λ_max_) recorded by microspectrophotometry in isolated rhabdoms is invariably 20–30 nm red-shifted in “lake” compared with “sea” populations. The dichotomy holds across species, major opsin lineages and light environments. Chromophore exchange from A1 to A2 (retinal → 3,4-didehydroretinal) is a well-known mechanism for red-shifting visual pigments depending on environmental conditions or stages of life history, present not only in fishes and amphibians, but in some crustaceans as well. We tested the hypothesis that the lake/sea dichotomy in *Mysis* is due to the use of different chromophores, focussing on two populations of *M. relicta* from, respectively, a Finnish lake and the Baltic Sea. They are genetically very similar, having been separated for less than 10 kyr, and their rhabdoms show a typical lake/sea difference in λ_max_ (554 nm vs. 529 nm). Gene sequencing has revealed no differences translating into amino acid substitutions in the transmembrane parts of their opsins. We determined the chromophore identity (A1 or A2) in the eyes of these two populations by HPLC, using as standards pure chromophores A1 and A2 as well as extracts from bovine (A1) and goldfish (A2) retinas. We found that the visual-pigment chromophore in both populations is A1 exclusively. Thus the spectral difference between these two populations of *M. relicta* is not due to the use of different chromophores. We argue that this conclusion is likely to hold for all populations of *M. relicta* as well as its European sibling species.

## Introduction

The crustacean genus *Mysis* encompasses a large number of species and populations living in marine, brackish-water and fresh-water environments. Repeated changes of habitats and light environments have occurred at both inter- and intraspecific levels on time scales ranging from millions to a few thousands of years [1]. This makes *Mysis* an attractive clade for studying rates and modes of visual evolution in relation to photic environment. Especially, a circumboreal group of “glacial relict” sibling species has yielded much insight into the roles of adaptations vs. phylogenetic constraints on shorter time scales [2], [3], [4], [5], [6]. Comparing single-rhabdom absorbance spectra with opsin gene phylogeny in nine populations of three European glacial-relict species (*M. relicta*, *M. salemaai*, *M. segerstralei*) from Scandinavian lakes and the Baltic Sea, ref. [7] concluded that differences in spectral absorbance show no clear correlation with either species, major opsin gene lineages, or current light environments. Instead, there is a consistent spectral dichotomy between “lake” and “sea” populations, such that spectral absorbance in all lake populations studied (n = 5) peaks at significantly longer wavelengths (range of within-population mean λ_max_ = 554–562 nm) than in any of the sea populations (λ_max_ = 521–535 nm; n = 4). If encoded by the opsin, this would suggest the unlikely scenario that parallel lake/sea divergences have occurred several times independently on quite different genetic backgrounds and time scales, even though there would often have been no evident advantage in terms of visual function (analyzed in terms of quantum catch and conceptual signal-to-noise ratio in “lake” and “sea” environments as described by Jokela-Määttä et al., 2007). Moreover, inter- and intraspecific spectral differences could not be consistently explained by amino acid substitutions in the opsins, whether in residues implicated in spectral tuning of vertebrate pigments [9], [10], [11], [12] or any other loci [7].

However, the absorbance spectrum of a visual pigment is determined not by the opsin alone, but by its interaction with the covalently bound cofactor, the chromophore. The use of varying proportions of two alternative chromophores, retinal (A1) and 3,4-didehydroretinal (A2), allows many fishes and amphibians to tune their visual pigments on a physiological time scale in response to environmental and physiological factors related to, e.g., seasons [13], [14] or stages of life history [15], [16]. Targeting of different A1 : A2 ratios to different regions of the retina or to different cell classes allows selective spectral tuning of different parts of the visual field [17] or of different photoreceptor types [18], [19]. For A1 pigments with λ_max_ around 500–550 nm, replacing A1 by A2 will red-shift λ_max_ by 20–50 nm, quite appropriate to account for the lake-sea dichotomy in *Mysis* [20], [21], [22]. The A1 : A2 system is found also in several fresh-water crustaceans [23], [24], and can be physiologically regulated by light and temperature just like in fishes and amphibians [25]. We therefore decided to test the hypothesis that the lake/sea dichotomy in *Mysis* is due to differential use of chromophores A1 and A2.

We focussed on a much-studied lake-sea pair of Finnish *M. relicta* populations, which are genetically very similar [2]. At the end of the last glaciation (ca. 8000 years ago: [26]), one population, here denoted L_P_, was entrapped in what is now the deep brown Lake Pääjärvi. The other population, here denoted S_P_, lives in the Baltic Sea at the SW coast of Finland (in Pojoviken Bay). Light transmission in the two water bodies peaks around 680 nm and 575 nm, respectively, but in Lake Pääjärvi very little light of any kind remains at the depths inhabited by the L_P_ animals [3]. The two populations show the characteristic lake/sea difference in spectral sensitivity, as the absorbance maxima of isolated rhabdoms measured by MSP lie at λ_max_(L_P_)≈554 nm and λ_max_(S_P_)≈529 nm [5]. There is a correlating difference in the activation energies of their visual pigments [27], [28], [29], confirming that the spectral difference reflects a real difference of the visual-pigment molecules and is not due to effects of, e.g., screening pigments or bleaching products. Yet, no differences have been found in the conceptual amino acid sequences of their opsins as derived from genomic DNA sequences covering the parts of the genes that code for the transmembrane parts of the opsin (unpublished results of J. Pahlberg and L. Kerosuo-Pahlberg; see [7]).

## Materials and Methods

### Ethical Statement

*Mysis relicta* is not an endangered species, but on the contrary, the most common macrocrustacean in Finnish waters. Under Finnish legislation, no permit is needed for the sampling of invertebrates. The coordinates of the sampling locations for the S_P_ and L_P_ animals are, respectively, N 59°59.90′ E 23°27.35′ and N 60°00.09′ E 23°27.52′. The former area (Pojoviken Bay) is a public shipping lane operated by the Finnish Transport Agency. Scientific study is part of the governance of the area; i.e., sampling for scientific purposes is not only allowed, but part of the intended use of the area. The latter area (Lake Pääjärvi) is a public water body, which has served as a limnological study area of the University of Helsinki since 1953. Quite regardless of any of the aforementioned conditions, Finnish legislation guarantees public access according to the general principle of “everyman’s right” (common rights) to all areas regardless of ownership (private/state/municipal), unless explicit and precisely specified regulations apply (which is not the case here). Common rights include unrestricted sampling of such invertebrate species that are not defined as endangered. The land-owner’s permission is never required for these purposes.

#### Goldfish

Five Ryukin goldfish (*Carassius auratus*) were bought on the day of the experiment in a pet shop in Moscow, where they were kept in aquaria under room temperature in standard conditions. Experiments were performed in compliance with the European Community Council Directive 2010/63/EU and the Institute of Biochemical Physics of the Russian Academy of Sciences policy. The fish were sacrificed by an overdose of anaesthetic by placing them in a solution of MS-222 (500 mg/l tricaine methanesulfonate, Sigma-Aldrich). No manipulations were carried out on the fish before sacrifice. No IACUC approval was obtained specifically for this study. Experiments on tissue isolated from animals that have been sacrificed in compliance with officially accepted procedures, without previous manipulations, do not require specific permission under Russian or EU law.

#### Cattle

Fresh bovine eyes (*Bos taurus*) were purchased from a meat processing factory (“Ramensky trade house”, Krasnoarmeiskaia st.131, 140109, Ramenskoe, Moscow region, Russia). The work was done with the permission of the slaughterhouse to use these bovine eyes for scientific research.

### Chemicals

The reagents N-Bromosuccinimide, N-phenylmorpholine, and sodium sulphate were of biochemical or reagent grade purity, obtained from Sigma-Aldrich (USA) and Component-Reactive Ltd (Russia). Solvents hexane, ethyl acetate and methanol of HPLC grade purity were purchased from Biosolve (The Netherlands). Tetrahydrofuran, dichloromethane and diethyl ether were obtained from Russian commercial suppliers and purified by distillation before the experiments. All*-trans*-retinal (A1) was obtained from Sigma-Aldrich (USA). Plastic 1-ml microcentrifuge tubes and disposable Pasteur pipettes from Eppendorf (Germany) were used. All reactions with reagents sensitive to oxygen and moisture were carried out in dry argon atmosphere. In the experiments with photosensitive compounds, care was taken to protect the samples from light by means of aluminium foil wrapped around the equipment.

### Mysis relicta: Animals and Preparation

#### Animals

The S_P_ animals were caught in daytime in July and August from a depth of about 20 m in Pojoviken Bay with an epibenthic sledge ending in a plastic bag. The L_P_ animals were caught in Lake Pääjärvi from a depth of 60–75 m by a vertical net ending in a cod-end, also in daytime, but care was taken to protect them from strong light exposures. The animals were transferred to plastic bags in styrofoam boxes containing water just previously collected from the same depth to minimize shocks from sudden changes in temperature or other factors. The animals were transported in dark containers in oxygen-rich water held at the temperature of the sampling locality to Tvärminne Zoological Station (University of Helsinki), where they were kept in dark aquaria at 4°C before examination.

#### Preparation of samples

The animals were decapitated and the eye stalks were cut under weak white microscope illumination, just strong enough to enable dissection under natural viewing. All the following steps were carried out in a room lit only by dim daylight (with curtains drawn). The eye samples were put into a glass homogenizer containing 2 ml of bi-distilled water and thoroughly homogenized. The homogenate was transferred to a round-bottom flask and treated with 2 ml of dichloromethane, after which the organic layer was separated with a 10-ml separating funnel or a Pasteur pipette. The extraction was repeated twice, and the organic layers were combined. The organic extract was dried over sodium sulphate, purified by chromatography on a silica gel microcolumn (mesh 40, Merck) with dichloromethane as the eluent, and the solvent was removed under reduced pressure. The dried extract was dissolved into the eluting system for subsequent HPLC analysis.

### Synthesis of All-trans-3,4-didehydroretinal (A2)

All*-trans*-retinal (500 mg, 1.76 mmol) was dissolved in 30 ml tetrahydrofuran (THF) and the solution was cooled on an ice bath. Then 780 mg (4.40 mmol) of N-bromosuccinimide dissolved in 5 ml THF was added under intensive stirring, and the reaction mixture was stirred at 0°C for 30 min. The reaction was monitored by thin-layer chromatography (TLC) on Kieselgel 60F_254_ precoated plates (Merck (Germany)) in a hexane : ethyl acetate (4∶1) mixture (R_f_ of the product was near 0.05). N-phenylmorpholine (570 mg, 3.52 mmol) was added to the reaction mixture, and the mixture was stirred overnight at a temperature below 0°C. The reaction was monitored by TLC (R_f_ of the product was near 0.55). When the reaction was complete, the mixture was evaporated and re-dissolved in 100 ml diethyl ether. The organic layer was washed with 1 M HCl (50 ml×2) and then water (50 ml×3). The combined extracts were dried over sodium sulphate and the solvent was removed under reduced pressure. The crude residue containing 3,4-didehydroretinal was pre-purified by flash-column chromatography on Kiеselgel 60 (Merck, (Germany)) to obtain fractions enriched with A2. A mixture of petroleum ether and ethyl acetate was used as the eluent, where the percent of ethyl acetate was raised from 0 to 10. The final 3,4-didehydroretinal product was obtained by preparative HPLC (model Smartline 1000, Knauer, Germany, silica gel column 5 µm, 20×250 mm, YMC, Japan). The eluent system was hexane : ethylacetate (90∶10, (v/v)) with the addition of 100 µl absolute methanol for 1 L eluent mixture, and flow rate 7.0 ml/min. The monitoring wavelength was 370 nm. The yield of pure 3,4-didehydroretinal was 227 mg (47%), R_f_ 0.55 (hexane : ethyl acetate (4∶1)), ε 43000 (λ_max_ 398 nm), 58–59°C. The structure of the prepared compound was confirmed by ^1^H-NMR spectroscopy and the absorbance spectrum, peaking in the UV, was recorded on a Shimadzu UV – VIS-2401PC spectrophotometer (Japan) in quartz cuvettes with thickness 10 mm. The physical-chemical properties of the synthesized 3,4-didehydroretinal complied with literature data [30], [31].

### Preparation of Biological Reference Samples from Vertebrate Retinas

Biological reference samples were prepared from retinas of vertebrate species agreed to contain “pure” chromophore, either A2 (Ryukin goldfish, see [32]) or A1 (bovine; no mammals have A2). Below, the preparatory procedures are described.

#### Goldfish

On the day of the experiment, 5 Ryukin goldfish (*Carassius auratus*) were bought in a pet shop, where they were kept in aquaria under room temperature in standard conditions. The fish were sacrificed by an overdose of anaesthetic by placing them in a solution of MS-222 (500 mg/l tricaine methanesulfonate, Sigma-Aldrich). The retinas from the 5 fish were then isolated, placed in a glass homogenizer containing 2 ml of bidistilled water, and thoroughly homogenized following the same procedures as for the *Mysis* samples. To favour formation of the all-*trans*-isomer, dissection and isolation of the retinas was done in full daylight. Subsequent steps were carried out under weak light.

#### Cattle

Fresh bovine eyes (*Bos taurus*) were obtained from a meat processing factory in Moscow. The retinas were isolated within 3 h after the death of the animal. Rod outer segments were harvested with a modified version of the method of preparative centrifugation in a sucrose density gradient [33]. To drive most of the chromophore into the all*-trans*-state, the rod outer segment suspension from 10 eyes was exposed to strong white light for 3 min (150 W incandescent lamp KGM 24–150; 400–700 nm with a heat filter). It was then placed in a glass homogenizer containing 2 ml of twice-distilled water and thoroughly homogenized, following the same procedures as for the *Mysis* samples. All these procedures were carried out under weak light.

### HPLC Analysis of Retinal Derivatives

Analysis was performed on a HPLC system (model Smartline 1000, Knauer (Germany)) with detector K-2500 with variable wavelength. The detection wavelength used was 370 nm. Optimal analytical separation of A1 and A2 retinal derivatives was achieved with two sequentially linked columns (Silica 7 µm, 250×4.6 mm (IBM Instruments, USA) and Kromasil 5 µm, 250×4.6 mm), using as eluent hexane – ethyl acetate (7% v/v) with the addition of absolute methanol (100 µl for 1 L mixture) and flow rate 1.0 ml/min.

## Results

**Figure 1:**
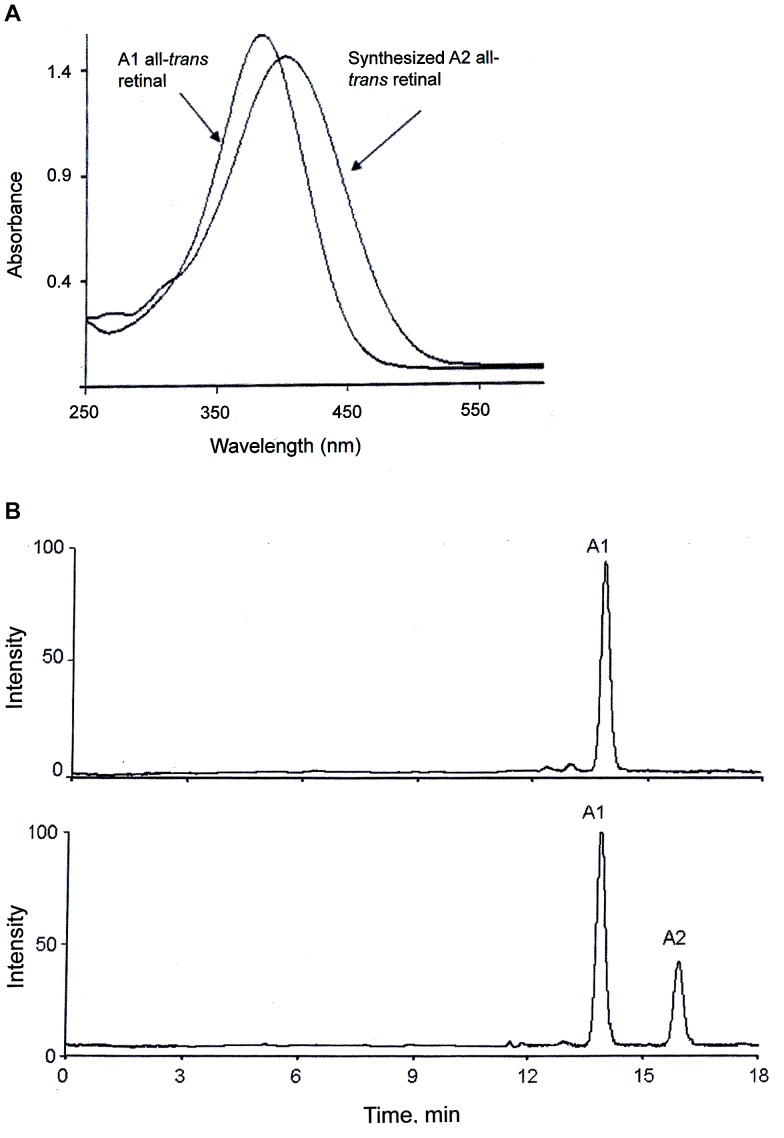
Characterization of the chromophore standards used: all-*trans*-retinal (A1) from Sigma-Aldrich (USA) and all-*trans*-3,4-didehydroretinal (A2) synthesized as described in the Materials and Methods section. (A) UV-VIS absorbance spectra of the A1 and A2 standards in methanol. (B) HPLC analysis of the A1 standard (top) and a mixture of the A1 and A2 standards (bottom). (Conditions: column Silica gel (7 µm, 4.6×250 mm (IBM)), isocratic mode, 8% diethyl ether in hexane (v/v), flow rate 1 ml/min; monitoring at 370 nm.).

The absorbance spectra of our A1 and A2 standards, i.e., the commercial all-*trans*-retinal (A1) and the synthesized all-*trans*-3,4-didehydroretinal (A2), are shown in Fig. 1 A. The spectra, peaking at 380 and 401 nm, respectively, are in good agreement with literature data [30], [31]. Fig. 1 B shows HPLC records for these standards, the top chromatogram for A1 alone, and the bottom chromatogram for a mixture of A1 and A2. Under the HPLC conditions we used, the peaks corresponding to A1 and A2 are obviously well separated.

**Figure 2:**
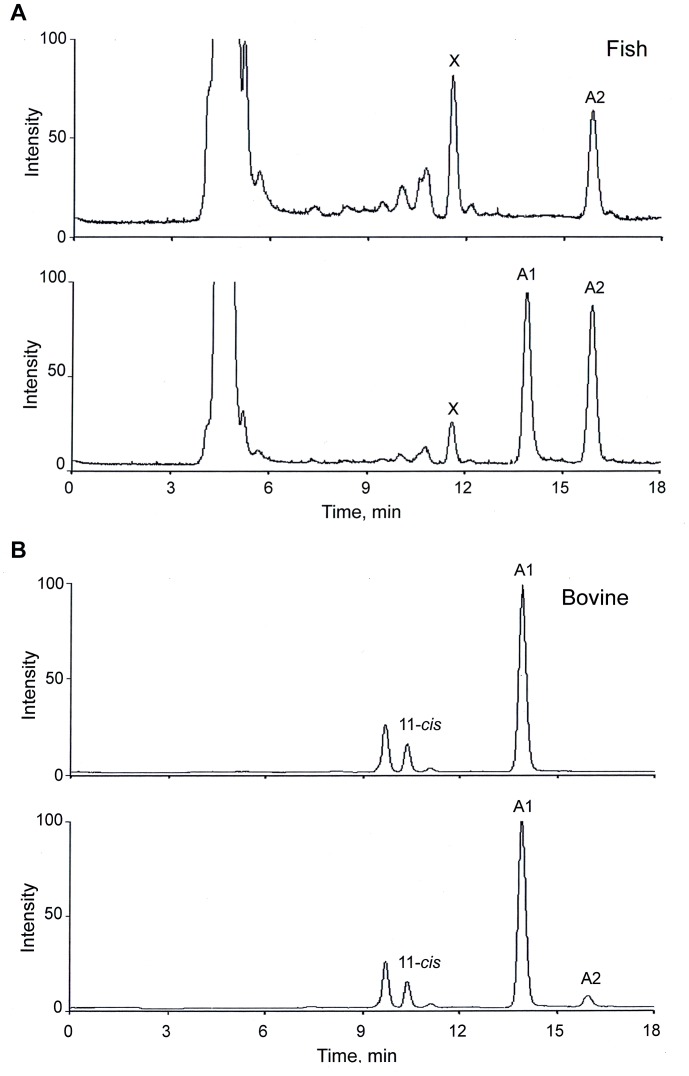
Comparative HPLC analysis of natural chromophores prepared from extracts of goldfish and bovine retinas (see Materials and Methods section) and the A1 and A2 standards. HPLC conditions as in Fig. 1. (A) Goldfish preparation (top chromatogram) vs. mixture of goldfish preparation and standards A1 and A2 (bottom chromatogram). The peak marked X is likely to correspond to the 11-*cis*-isomer of A2. (B) Bovine preparation (top chromatogram) vs. mixture of bovine preparation and standards A1 and A2 (bottom chromatogram).

Fig. 2 shows results of control experiments confirming that the A1 and A2 standards behave identically to extracts of visual-pigment chromophores from animal eyes under the HPLC conditions used. The animal samples were prepared from eyes of Ryukin goldfish, representing pure A2, and bovine eyes, representing pure A1 as described in the Materials and Methods section. In Fig. 2 A, the HPLC analysis result of the goldfish extract (top chromatogram), is compared with that of a mixture of the same extract and the standards A1+ A2 (bottom chromatogram). The main difference is the appearance of a prominent new peak (consistent with A1) in the bottom chromatogram, not present in the top chromatogram. In addition, there is a relative growth of the goldfish chromophore peak, consistent with coincidence of the goldfish peak with that for the A2 standard. In Fig. 2 B the same comparison is done for the bovine extract. Here, the addition of the A1+A2 standard mixture to the extract is associated with a new peak in the A2 position (bottom chromatogram), not present in the bovine extract alone (top chromatogram). These results are of course as such completely unsurprising. However, they serve to confirm that our all-*trans* standards and corresponding natural chromophore from retinal extracts behave identically under our HPLC protocol.

**Figure 3:**
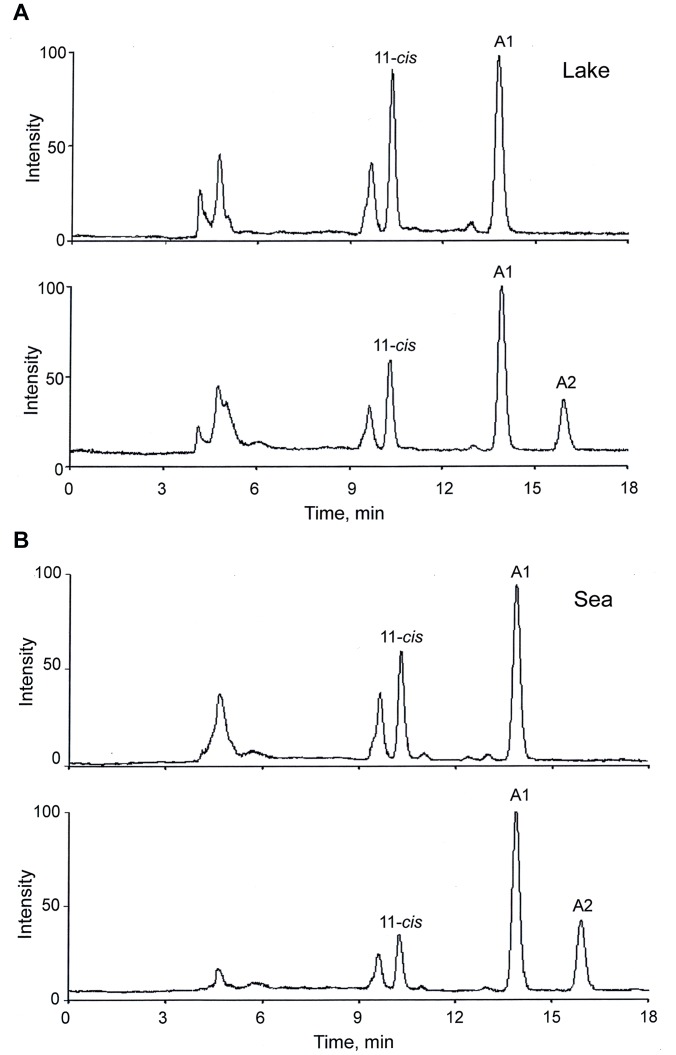
Comparative HPLC analysis of chromophore extracts from eyes of *Mysis relicta* from populations L_P_ (lake) and S_P_ (sea). HPLC conditions as in Figs. 1 and 2. (A) L_P_ preparation (top) vs. mixture of L_P_ preparation and standards A1 and A2 (bottom). (B) S_P_ preparation (top) vs. mixture of S_P_ preparation and standards A1 and A2 (bottom).

We were also able to identify 11-*cis* isomer of A1 in extracts from bovine rod outer segments using the standard 11-*cis* isomer of A1 prepared according to Pat. RU No 2417983 [34] (chromatograms with 11-*cis* standard not shown). These peaks are labelled accordingly in Fig. 2 B and Figs. 3 A and B, and consistent with these, we tentatively attribute the peak marked X in Fig. 2 A to the 11-*cis*-isomer of the goldfish chromophore (A2).

Fig. 3 illustrates the results of our main experiments to identify the visual pigment chromophore in the eyes of the L_P_ (panel A) and S_P_ (panel B) populations of *M. relicta*. The rationale was the same as in the goldfish and bovine experiments described above. The HPLC chromatograms for extracts from the eyes of the respective population (top chromatograms in panels A (L_P_) and B (S_P_)) are compared with the HPLC chromatograms for the same extracts with the A1+A2 standard mixture added (bottom chromatograms in panels A and B). In both panels (i.e., both populations), it is obvious that the only major change brought by the addition of the A1+A2 standard mixture is the appearance of a new peak in the A2 position, not present in the eye extracts alone. This indicates unambiguously that the eyes of L_P_ and S_P_ *M. relicta* contain no A2 chromophore. In other words, the chromophore of both populations is pure A1.

## Discussion

The *M. relicta* populations L_P_ and S_P_ offer an attractive pair for addressing the origins of the consistent spectral-sensitivity difference between *Mysis* populations trapped in fresh-water lakes and those living in brackish water. They show a typical lake/sea shift (25 nm) in the spectral absorbance of single rhabdoms, yet they are genetically very similar, which constrains possible explanations. No differences have been found in the conceptual amino acid sequences of their opsins as derived from genomic DNA sequences covering by now practically the whole transmembrane parts of the opsin (unpublished results of J. Pahlberg and L. Kerosuo-Pahlberg; see [7]). Admittedly, invertebrate opsins might have “unconventional” tuning sites e.g. in the cytoplasmic domains that have not yet been sequenced (for structural differences between a G_q_-coupled opsin and vertebrate opsins, see [35]. Yet, different chromophore usage offers an obvious and parsimonious hypothesis to account for the occurrence of similar spectral differences on the quite different genetic backgrounds of three *Mysis* species. Chromophore exchange A1 ↔ A2 is used not only in vertebrates, but also in some crustaceans [23], [24], [25] to produce spectral shifts of the right order of magnitude. Thus the aim of the present study was to test the hypothesis that the red-shift of “lake” compared with “sea” *M. relicta* has been achieved by replacing chromophore A1 by A2.

The results of our experiments are very clear. HPLC analysis of chromophore samples from the two populations against A1 and A2 standards revealed no A2 in either the L_P_ or the S_P_ animals, whereas A1 was prominent in both. Thus the hypothesis that the difference in spectral sensitivity of these two populations of *M. relicta* is due to a chromophore difference can be definitely rejected.

To what extent can this conclusion be generalized to other populations of *M. relicta* and its European sibling species, where similar lake/sea spectral differences have been observed? As we have here studied only one lake and one sea population of one species, of course there remains the possibility that differences could in other cases still be due to chromophore differences. However, if a functional A1–A2 system were present and biologically important in other populations of *M. relicta*, and/or in *M. salemaai* and *M. segerstralei*, it is very hard to understand why the particular population pair studied here should have lost it and had to reinvent a similar spectral difference by some other means. It thus seems justified to think that chromophore exchange is not the mechanism underlying the lake/sea spectral difference in any of the European glacial-relict species studied in refs. [5], [7].

Our identification of the visual-pigment chromophore as pure A1 means that we are still left in ignorance regarding the molecular or cellular origins of the spectral dichotomy of lake and sea populations. Thus we have to consider alternative possibilities. The presence of very similar differences across species and populations, with speciation and separation histories covering times from millions of years down to a few thousands of years and involving repeated transitions between “lake” and “sea” conditions, may be explained along at least two quite different lines: (i) A similar lake/sea difference may have been independently reinvented over and over again by hitherto unknown tuning mechanisms on different genetic backgrounds and different time scales; or (ii) The difference may arise phenotypically from a shared “reaction norm” [36] - a developmental switch sensitive to some environmental factor(s) differing between lake and sea environments and conserved from the shared deeper history of the clade.

The latter alternative seems much more likely. Phenotypic plasticity of spectral absorbance can be achieved not only by variable chromophore proportions, but also by variable expression of several opsins, as extensively studied in fishes (e.g. [37], [38], [39], [40], [41], [42], [43], [44]). These investigations present a very rich picture of selective adjustment of the spectral properties of specific cell classes in response to life-history factors. This line of reasoning is encouraged to some extent by the recent finding that rhabdoms of *M. relicta* contain two spectrally different types of photoreceptors [45].

Whatever the “final pathway” that implements a lake/sea switch in spectral sensitivity during development, however, it remains a major (and separate) question what environmental cues the switch responds to. Earlier notions that the lake/sea differences in spectral sensitivity are “adaptive” [46], [3], [5] in terms of quantum catch or signal-to-noise ratios achieved in the different light environments cannot be generally upheld [7, cf. 8].

In the North American sibling species *M. diluviana*, formerly included in the species *M. relicta*, studies are restricted to “lake” populations, and nothing can be said at the moment about possible systematic variation of visual pigments with habitat. Consistent with our present results for European members of the *M. relicta* species group, all populations seem to use the A1 chromophore. The *M. diluviana* populations in Cayuga Lake (New York) and Lake Ontario have an A1 pigment peaking at 520 nm [47], [48]. It should be noted, however, that chromophore identification in these studies was based on the shape of single-rhabdom absorbance spectra, a method fraught with uncertainties (see below). In the populations of Pend Oreille and Hayden Lake (Idaho), single-rhabdom spectra showed a λ_max_ variation range of 495–529 nm (n = 27) with mean λ_max_ ≈ 510 nm [5]. At least at the low end of this range, the shapes of spectra were consistent with pure A1. As such, spectral differences between *M.diluviana* and *M. relicta* and *M. salemaai* with the same A1 chromophore is not surprising, since several non-conserved amino acid substitutions have been indicated by genomic opsin DNA sequencing [7].

We would finally like to point out that the use of pure A1 chromophore by *M. relicta* makes functional sense in light of what is known about spontaneous (thermal) activation of visual pigments. A2 pigments are thermally much less stable than their A1 pairs [32], implying that they generate much stronger intrinsic “dark” noise due to randomly occurring photon-identical events, impairing the detectability of real light [49], [50]. Previously, the chromophore of all three European glacial-relict *Mysis* species were tentatively identified as A2, as MSP spectra recorded from single rhabdoms were best-fitted by the broader spectral templates for A2 visual pigments [5,7, cf. 51]. It always seemed puzzling why *Mysis* populations should use the noisy A2 chromophore, as many of them spend at least part of their lives in very dark environments. The misidentification was due to the fact that the *shape* of recorded spectra is not a very reliable indicator of chromophore content (see [19]). Quite regardless of variations in technical quality, recorded spectra may be broadened (or indeed narrowed) by several factors, e.g. various kinds of screening as well as the presence of bleaching products or mixtures of visual pigments [45].
